# Membrane Interactions of S100A12 (Calgranulin C)

**DOI:** 10.1371/journal.pone.0082555

**Published:** 2013-12-18

**Authors:** Assuero F. Garcia, José L. S. Lopes, Antonio J. Costa-Filho, Bonnie A. Wallace, Ana P. U. Araujo

**Affiliations:** 1 Instituto de Física de São Carlos, Universidade de São Paulo, São Carlos, SP, Brasil; 2 Departamento de Física, Faculdade de Filosofia Ciências e Letras de Ribeirão Preto, Universidade de São Paulo, Ribeirão Preto, SP, Brasil; 3 Institute of Structural and Molecular Biology, Birkbeck College, University of London, London, United Kingdom; Griffith University, Australia

## Abstract

S100A12 (Calgranulin C) is a small acidic calcium-binding peripheral membrane protein with two EF-hand structural motifs. It is expressed in macrophages and lymphocytes and highly up-regulated in several human inflammatory diseases. In pigs, S100A12 is abundant in the cytosol of granulocytes, where it is believed to be involved in signal modulation of inflammatory process. In this study, we investigated the interaction of the porcine S100A12 with phospholipid bilayers and the effect that ions (Ca^2+^, Zn^2+^ or both together) have in modifying protein-lipid interactions. More specifically, we intended to address issues such as: (1) is the protein-membrane interaction modulated by the presence of ions? (2) is the protein overall structure affected by the presence of the ions and membrane models simultaneously? (3) what are the specific conformational changes taking place when ions and membranes are both present? (4) does the protein have any kind of molecular preferences for a specific lipid component? To provide insight into membrane interactions and answer those questions, synchrotron radiation circular dichroism spectroscopy, fluorescence spectroscopy, and surface plasmon resonance were used. The use of these combined techniques demonstrated that this protein was capable of interacting both with lipids and with ions in solution, and enabled examination of changes that occur at different levels of structure organization. The presence of both Ca^2+^ and Zn^2+^ ions modify the binding, conformation and thermal stability of the protein in the presence of lipids. Hence, these studies examining molecular interactions of porcine S100A12 in solution complement the previously determined crystal structure information on this family of proteins, enhancing our understanding of its dynamics of interaction with membranes.

## Introduction

The superfamily of calcium binding proteins (CaBPs), also called EF-hand proteins [1], is involved in several physiological functions such as motility, cell growth and differentiation, cell cycle regulation, secretion, Ca^2+^ homeostasis, regulation of enzyme activity, protein phosphorylation, organization of cytoskeleton, and blood coagulation [2]. There are two general classes of EF-hand proteins: the Ca^2+^ sensors, which act in Ca^2+^ signal transduction as a result of an increase in Ca^2+^ concentration, and the Ca^2+^ buffers, which act to modulate the Ca^2+^ signal, maintaining a safe concentration of this ion in the cytosol [3].

S100 proteins [4] are a major subfamily of the EF-hand sensor proteins, with low molecular mass (10–14 kDa), 25–65% sequence identities, and three-dimensional structures that are highly conserved across species. They are exclusively expressed in vertebrates, where their expressions are tissue- and cell-type specific [5].

S100 proteins contain two motifs that bind Ca^2+^ per molecule [6]. One site is a canonical EF-hand with twelve amino acids flanked by two helices with a C-terminus that shows high affinity for Ca^2+^. The second site is a ‘pseudo-EF-hand’ or ‘S100-specific EF-hand’. It consists of a fourteen amino acid consensus sequence motif with an atypical backbone conformation compared to the usual EF-hand, and includes a number of well-conserved basic residues [7]. These features result in this EF-hand having a lower affinity for Ca^2+^ due to the missing side chain oxygen atoms of Asp or Glu that are essential for high affinity binding. The EF-hands are linked to each other by a hinge region, which exhibits low sequence similarity among S100 proteins.

Besides Ca^2+^, a number of S100 proteins can also bind Zn^2+^ or Cu^2+^. The interactions with divalent metal cations modulate their functional properties, inducing changes in their affinity for interaction partners and promoting homo- or hetero-oligomerization. Comparisons of apo- and holo-protein crystal structures [8] indicate that Ca^2+^ binding generally produces helix rearrangements within each subunit of the dimer, resulting in the exposure of hydrophobic surfaces (one in each monomer). These surfaces are comprised of residues present in the hinge region and are involved in target protein recognition [9]–[11].

S100A12 is a member of the S100 family that is expressed in macrophages, endothelium and lymphocytes, being highly up-regulated in several human inflammatory diseases [12], including Crohn's disease, rheumatoid arthritis, cystic fibrosis, Kawasaki disease, and other inflammatory states [13], [14]. It exists under physiological conditions as a non-covalent antiparallel dimer held together by hydrophobic interactions. S100A12 binds two calcium ions per subunit and one zinc ion with high affinity. Moreover, zinc binding increases both calcium binding and target protein binding affinities [15]. S100A12 is highly abundant in the cytosol of granulocytes [16], but it is able to translocate to membranes following the interactions with calcium [12].

Although S100A12 specific functions have not yet been fully elucidated, it has been shown that interactions between S100 proteins and membranes are present in different physiological responses, such as inflammatory processes, neutrophil chemotaxis and cell adhesion [17]. In particular, S100A12 is involved in induced migration of monocytes [18]. It is known that the folding and stability of S100A12 can be modulated by the presence of the metal ions [19]. Moreover, the cell location of S100A12 has been shown to be calcium dependent, with an elevated calcium concentration being responsible for the protein transfer from the cytosol to the membrane fraction [20]. It can thus be concluded that states of S100A12 when bound to ions and/or membranes are part of the protein activity. Hence, studies that can address how the protein-membrane interaction is affected by the presence of divalent ions are important for unraveling basic aspects of the protein participation in these processes.

Recently, a thermodynamic study with recombinant porcine S100A12 demonstrated that its structural stability was increased in the presence of Zn^2+^ and Ca^2+^ [21]. However, the metal ions influence on the dynamics conformation and stability of S100A12 in the presence of membrane models has not been investigated so far. In the present study, synchrotron radiation circular dichroism (SRCD) spectroscopy was used to further investigate the intermolecular interactions relevant to S100A12 role as a peripheral membrane protein, notably were there different effects on its structure, binding and stability associated with different lipid head group types. SRCD spectroscopy, fluorescence spectroscopy, and surface plasmon resonance (SPR) were used to monitor secondary structural changes and thermal stability, tertiary structural changes, and binding affinities, respectively, to provide insights regarding S100A12-membrane interactions.

## Materials and Methods

### Protein expression and purification

Porcine S100A12 [Uniprot ID P80310] was produced as previously described [21]. Briefly, *E. coli* BL21(DE3) cells harboring the pET28a-S100A12 vector were grown at 37°C in Luria-Bertani medium containing kanamycin (30 µg/mL), then induced with isopropyl-β-D-thiogalactopyranoside (0.1 mM). Cell growth continued at 22°C. The cells were harvested by centrifugation and the cell pellets were stored at −20°C. Cells were resuspended in 20 mM Tris, 150 mM NaCl, pH 8.0 and lysed by sonication. Insoluble material was removed by centrifugation. S100A12 was purified on a Ni-NTA superflow column (QIAGEN). The column was washed with buffer and the target protein was eluted with 250 mM imidazole. The cleavage of His-tag from S100A12 was performed using 1 unit of thrombin (Sigma) to 250 µg of protein for 24 h at room temperature. The thrombin was further removed using a benzamine resin (GE Healthcare). Samples from each step were analyzed on SDS-PAGE (Suppl. Information). Protein samples were then dialyzed against the buffers used for SRCD, fluorescence and SPR studies. Protein concentration was determined by UV absorbance at 280 nm using the extinction coefficient ε_280_ = 19.940 M^−1^.cm^−1^calculated using ProtParam software [22] on Expasy server.

### Sequence alignment of S100A12 proteins

Alignment between the porcine S100A12 (Uniprot code P80310) and the human protein (Uniprot code P80511) was performed with protein BLAST program [23]. The 2Struc webserver [24] was used to quantify the secondary structure of human S100A12 in the apo- and holo-form.

### Steady state fluorescence spectroscopy

The intrinsic fluorescence emission spectra of S100A12 (5 µM) in 5 mM HEPES buffer (pH 7.4) were measured in the presence of large unilamellar vesicles (LUVs) of either 1,2-dipalmitoyl-sn-glycero-3-phosphocholine (DPPC) or 1,2-dipalmitoyl-sn-glycero-3-phosphoglycerol (DPPG), prepared using a 1∶100 protein∶lipid molar ratio, as previously described [25]. Measurements at 25°C were made using an ISS K2 spectrofluorimeter (ISS Fluorescence, Analytical and Biomedical Instruments, Illinois, USA) with excitation at 274 nm in a 1 cm pathlength quartz cuvette. Emission spectra were recorded over the wavelength range from 290 to 450 nm. Calcium chloride and/or zinc chloride were added in a single aliquot (5 µL) to yield a 1 mM solution.

### Synchroton Radiation Circular Dichroism Spectroscopy

The SRCD spectra of S100A12 (75 µM) in aqueous solution were collected over the wavelength range from 280 nm to 170 nm, using a 1 nm interval and a 2 s dwell time, at 25°C, in a cylindrical quartz cell (Hellma Ltd.) with a pathlength of 99 µm on the CD1 beamline located at the ISA synchrotron (University of Aarhus, Denmark). CDTools [26] software was used for data processing: the average of the three scans of the corresponding buffer baseline (10 mM sodium phosphate pH 7.0) was subtracted from the average of three scans of the sample, smoothed with a Savitky–Golay filter, calibrated with a spectrum of camphour sulphonic acid measured at the beginning of the data collection, and converted to delta epsilon units using a mean residue weight of 118. Analyses of the secondary structural contents used the DichroWeb server [27], with database SP175 [28] and the algorithm ContinLL [29].

SRCD studies of S100A12 binding to the liposomes entailed incubating the protein (75 µM) for 10 min at 25°C with vesicles of DPPC and DPPG at a protein/lipid ratio of 1∶100 and 10 mM Tris, pH 8.0. Measurements were obtained as above except using a pathlength of 104.5 µm.

Thermal stability studies of S100A12 in both the presence and absence of liposomes and/or 1 mM Ca^2+^ and/or Zn^2+^ were performed over the temperature range from 5 to 85°C in 5°C increments, using a 5 min equilibration time at each temperature. Three repeat measurements were made at each temperature, and the first and last of these compared to ensure that thermal equilibrium had been achieved prior to the measurement at each temperature.

### Surface Plasmon Resonance (SPR)

The interaction of apoS100A12 with phospholipid bilayers in 10 mM HEPES (pH 7.4), 100 mM NaCl was analyzed by SPR on a BIAcore X (GE Healthcare). Initially, a solution of octyl β-D-glucopyranoside solution (40 mM) was used to clean the L1 sensorchip [30]. LUVs of DPPG and DPPC with average diameter of 100 nm were applied for 50 min, with a flow rate of 1 µL/min, at 25°C, to immobilize them on the sensorchip surface. The lipidic surface was washed by injections of 4 mM NaOH. The negative control of bovine serum albumin (1.5 µM) was applied to both cells. Increasing concentrations of S100A12 (ranging from 31 nM to 8 µM) in the HEPES buffer were injected onto the sensorchip, and the interaction with the phospholipid bilayer was monitored for 10 min at a flow rate of 20 µL/min. After this time, the running buffer alone was injected to monitor S100A12 dissociation from the lipid bilayer. The binding of S100A12 to the liposomes was followed in real-time by the sensorgrams resulting from the kinetics assays with the immobilized lipid bilayer on the L1 Sensorchip (GE Healthcare) and the protein injected at different concentrations. For each trial, the response of a control surface (cell with no lipids immobilized) was subtracted out to eliminate any nonspecific binding and refractive index changes due to buffer change.

The affinity constants were directly determined from the equilibrium binding responses over a range of protein concentrations (from 31 nM to 8 µM) by fitting the data to a Langmuir adsorption isotherm. The curve fitting was checked by residual plots and χ^2^. All data analysis was performed using BIAevaluation software (Biacore, version 4.1). The dissociation phase was fit to the integrated rate equation: 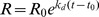, where R_0_ is the response at the start of the fit data, k_d_ is the dissociation rate constant, and t_0_ is the time at the start of the fit data. The association phase was fitted to the integrated equation: 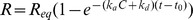, where C is the analyte (protein) concentration, and k_a_ is the association rate constant. The dissociation constant (K_d_) was then calculated from the equation: 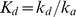 [31].

### Release of entrapped calcein from lipid vesicles

LUVs of DPPG, loaded with the fluorescent probe calcein (Sigma-Aldrich), were prepared by dissolving the phospholipid in a chloroform/methanol 2∶1 (v/v) mixture, followed by the solvent evaporation to yield a thin lipid film. The film was hydrated by the addition of a buffer containing 10 mM HEPES, pH 7.4, 10 mM NaCl and 35 mM calcein at 45°C, and vigorously agitated by vortexing. The vesicle suspension was freeze-thawed and then extruded through a Mini-extruder (Avanti Polar Lipids, Inc.). Non-encapsulated calcein was removed on a Sephadex G-65 column and eluted with 10 mM HEPES pH 7.4, 100 mM NaCl. Liposome concentrations were determined from organic phosphorus assays [32]. Fluorescence was measured using a 1 cm pathlength quartz cuvette, under continuous stirring, with excitation at 490 nm and emission at 520 nm, at 25°C. Increasing concentrations of S100A12 (from 1 to 9 µM) were added to LUVs (0.1 mM), and the kinetics of the release of calcein from was monitored as an increase in fluorescence intensity after 10 min. The percent of leakage was calculated according to the following equation:
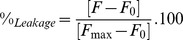
where 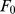 is the fluorescence intensity of the intact liposomes, *F* and 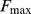 the respective intensities just before and after the addition of 10% (w/v) Triton X-100 to the cuvette (ie. before and after disruption of the LUVs).

## Results and Discussion

### S100A12 alignment and structure comparison

Human and porcine S100A12 exhibit a 75% sequence identity (Suppl. Information). According to their locations in the human enzyme, the canonical EF-hand will be located between residues 19 and 33, and the non-canonical one might be located between residues 62 and 74 in the porcine enzyme. The zinc binding sites are located at residues His16, Asp26, His86 and His90.

The secondary structures of human S100A12 calculated from the crystal structures consist of 63% of helix, 4% strand and 33% of other (disordered coil plus turn) in the apo-form (PDBID 2WCE), and 61% helix, 4% strand, and 35% other in the holo-form (PDBID 1E8A). The secondary structure content of the apo- and holo- forms of porcine S100A12 determined using SRCD spectroscopy (Table 1) were found to be 62% helix, 2% strand, and 36% other, versus 64% helix, 2% stand and 34% other, respectively, suggesting there is very little difference in the porcine structures at 25°C.

**Table 1:** Secondary structure content of human (calculated from crystal structures) and porcine S100A12 (determined from SRCD spectra).

		α-helix (%)	β-strand (%)	other (%)
Human	apo	63	4	33
	holo	61	4	35
Porcine	apo	62	2	36
	Ca^2+^	64	2	34
	Zn^2+^	64	2	34
	Ca^2+^/Zn^2+^	65	1	34
	DPPC	65	1	34
	DPPC+Ca^2+^	65	1	34
	DPPC+Zn^2+^	62	4	34
	DPPC+Ca^2+^/Zn^2+^	64	2	35
	DPPG	69	1	30
	DPPG+Ca^2+^	24	23	53
	DPPG+Zn^2+^	30	17	53
	DPPG+Ca^2+^/Zn^2+^	46	11	43

### Intrinsic Fluorescence Emission Spectroscopy

Porcine S100A12 has two tyrosine residues, one of which (Tyr18) appears in a conserved position when compared to the human S100A12 and is partially buried in the apo- and holo-forms of the crystal structure. In the holo-form of human S100A12, the second Tyr residue is slightly less exposed to the solvent than in the apo-protein, as described by Moroz et al. [33].

**Figure 1:**
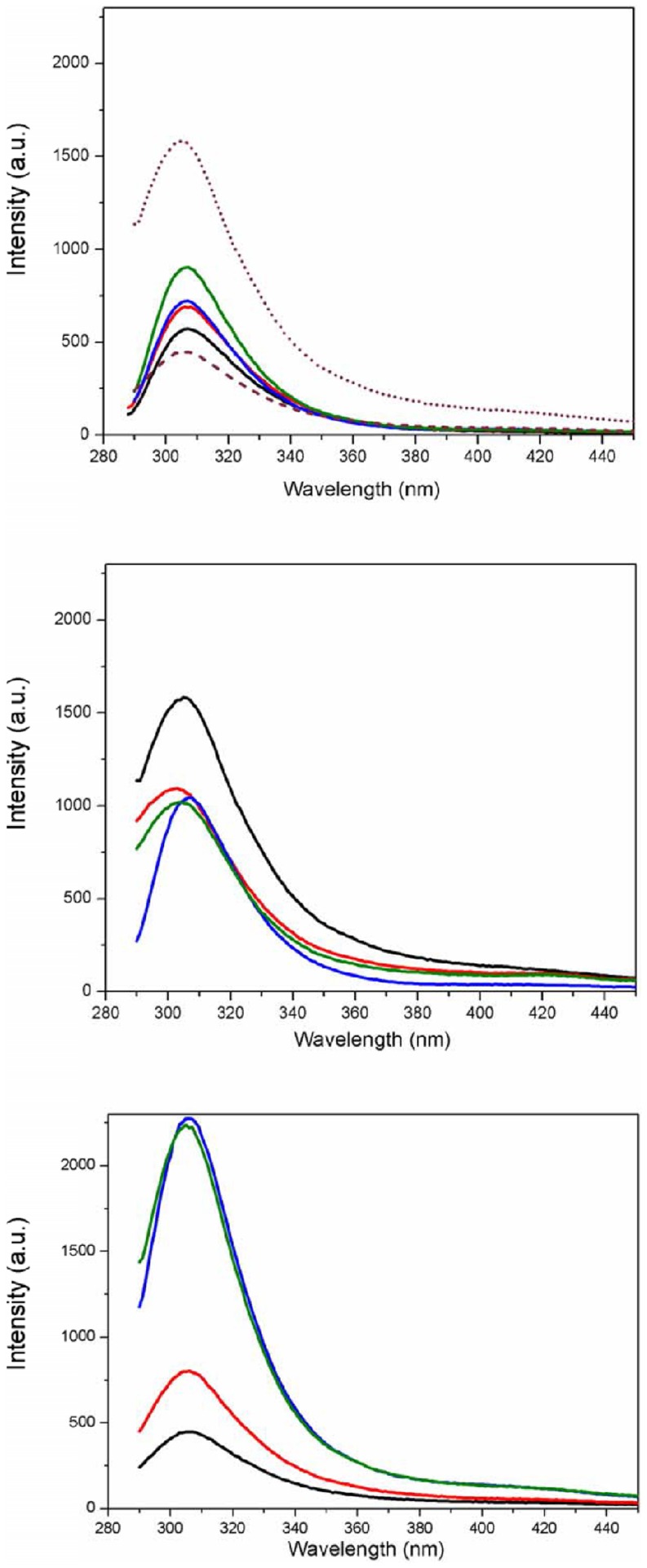
Fluorescence emission spectra of S100A12 (5 µM) a) in aqueous solution (black) and incubated with Ca^2+^ (red), Zn^2+^ (blue), both ions together (green), DPPC vesicles (dot grey), DPPG (dash grey) vesicles. b) in the presence of DPPC liposomes (black) and c) DPPG liposomes (black), in both cases with Ca^2+^ (red), Zn^2+^ (blue), or Ca^2+^/Zn^2+^ together (green).

A series of fluorescence emission spectra were measured to evaluate the binding of porcine S100A12 to liposomes in aqueous solution and the influence of Ca^2+^, Zn^2+^ and the mixture of both ions in that process. In aqueous solution the emission maximum occurred at 307 nm (Figure 1a) for the apo-protein. When either of the ions was added to the solution, an increase of ∼20% in its fluorescence intensity was observed. Moreover, when both ions were added together, the fluorescence intensity increase was even greater (∼60%). The calcium and zinc binding sites in the first EF-hand motif are located very close to the two Tyr residues. In porcine S100A12 the first Tyr residue (Tyr18) is located at the beginning of helix two (H2) and the second one (Tyr25) is part of the non-canonical EF-hand loop (loop 1). Upon Zn^2+^ and/or Ca^2+^ binding to S100A12 the microenvironments of both Tyr residues seem to be affected, which is also true in the presence of the vesicles only (Figure 1a). The S100A12 structural alterations taking place upon ion binding can be visualized in Fig. S12 (Suppl. Information).

Ion binding to S100A12 induced an enhancement of Tyr fluorescence quantum yield (Figure 1), showing that in the absence of ions the Tyr residues were located in different microenvironments. As already observed for other S100 members, the fluorescence enhancement must be related to changes in the environment of the Tyr involving the removal of quenching groups (such as COO- or CONH) [34]. The binding of calcium to S100A12 induced a drastic reorientation of helix 2 (Tyr18) and helix 3. As a consequence, a hydrophobic pocket was formed by helixes 3 and 4 and loop 2. Such pocket creates a redistribution of the surface charge that probably affects the region involved in target recognition (residues from Lys40 up to Thr50). This effect induced by the presence of Ca^2+^ is common to several members of S100 proteins. The exposition of a hydrophobic cleft upon Ca^2+^ binding is accompanied by physiological responses, related to recognition and interaction with molecular targets, such as proteins and other ligands [34]–[36].

The presence of both ions at their respective binding sites promotes conformational rearrangements within this microenvironment in the protein and also the displacement of water molecules from the region, which could account for the observed effect on the fluorescence emission. A similar, albeit larger, effect has been reported in calmodulin [37], in which an increase in fluorescence has been reported upon addition of Ca ions. It was suggested in that case that the spectral changes were due to one (Tyr138) of the two tyrosine residues present in its C-domain [38], which appears to be in a less polar environment when Ca^2+^ was bound to the protein.

The binding of S100A12 to the DPPC LUVs (Figure 1b) promoted a 2.8-fold increase in the fluorescence intensity, which could also be a consequence of the changes in the surroundings of one or both of the Tyr residues, probably due to the changes in its exposure to a less polar environment at the water/lipid interface of the bilayer. Preincubation of S100A12 with either Ca^2+^ or Zn^2+^ and then addition to zwitterionic liposomes resulted in an increase in the fluorescence intensity of approximately 50% when compared to that of the protein in the absence of the liposome (Figure 1b). Similar fluorescence intensity was observed when S100A12 was bound to both metal ions in the presence of DPPC, with an approximate 80% increase in the fluorescence intensity compared to the apo-protein.

In contrast, the fluorescence intensity of apo-S100A12 when incubated with DPPG liposomes was reduced (Figure 1c). The charged nature of this phospholipid surface could act as a fluorescence quencher for the Tyr residue, most likely the one that is exposed to the liposome interface. However, when S100A12 was bound to Ca^2+^ and incubated with the negatively charged liposomes, a 40% increase in fluorescence intensity was observed. This effect was enhanced substantially in the presence of Zn^2+^, with a 4-fold increase in fluorescence emission, suggesting that the holo protein is capable of binding more effectively to these lipids. These results suggest a synergy in the binding to the DPPG liposomes in the presence of the ions, resulting from a significant conformational change in the microenvironment of the Tyr residues that becomes more buried from the aqueous environment.

### SRCD Spectroscopic studies of molecular interactions

**Figure 2:**
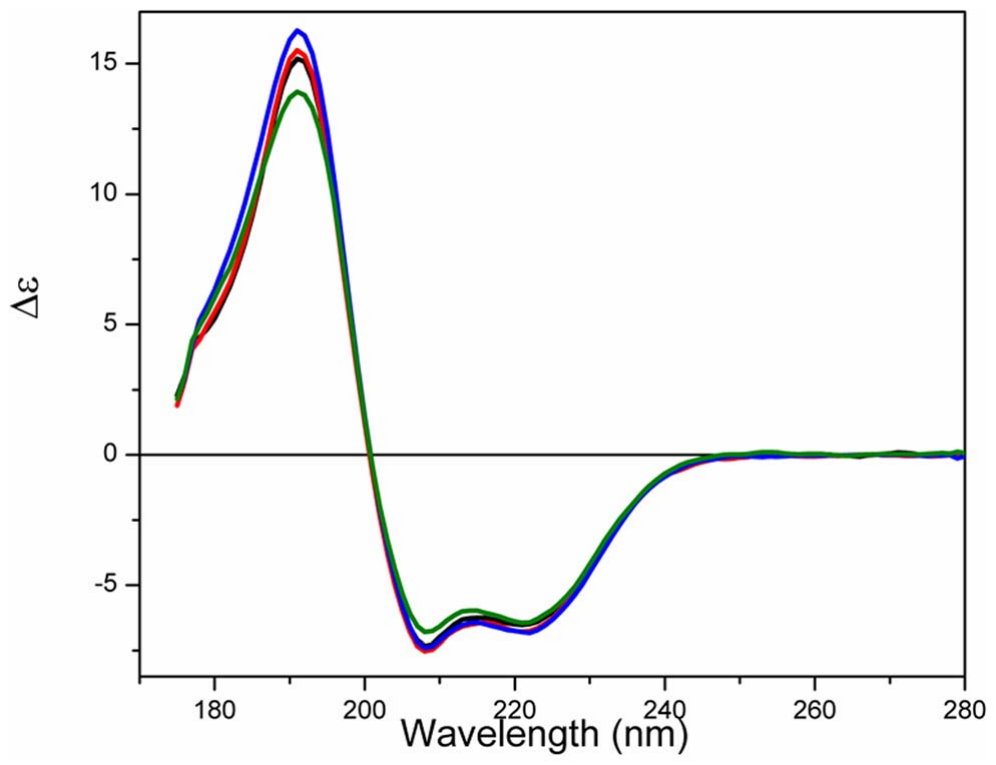
SRCD spectra of S100A12 (75 µM) in aqueous solution in apo-state (black), and in the presence of Ca (red), Zn (blue) and both ions together (green).

Conventional (lab-based) circular dichroism (CD) spectroscopy has been previously used [21] to examine S100A12 in solution with and without ions present. In this paper, the use of SRCD spectroscopy has extended the spectra to much lower wavelengths, enabling more accurate estimates of the secondary structure contents and clearer observation of structural changes. Furthermore, the high flux of the synchrotron light enhances the signal-to-noise levels of the measurements, thus overcoming the interference of both the lipids and the high salt concentrations in the buffers used to study this protein. Even though the NaCl absorbance is non-chiral, it is substantial in the far UV wavelengths, and hence diminishes the amount of light reaching the detector, thus making measurements noisier and ultimately preventing measurements below ∼210 nm. Because the information below this wavelength is critical for identifying secondary structures containing sheet and disordered structures, SRCD enables much more subtle conformational changes to be detected. The SRCD spectrum of apo-S100A12 (Figure 2) exhibits negative peaks at 222 nm and 208 nm, a positive maximum at 193 nm and a shoulder in the region of 180–185 nm. These are characteristics of a protein that is dominated by helical secondary structure (Table 1) but which has a significant amount of structure that is either in turns or unordered.

It has been reported that the EF-hand motifs can assume an open conformation after Ca^2+^ binding due to a conformational change attributed to the rearrangement of the helixes in the motif [39]. In S100B, a large rotation (∼90 degrees) of helix 3 is observed in the typical EF-hand domain (EF2) upon the addition of calcium [40]. Still for S100B, a comparison between apo- and Ca^2+^-bound forms indicates that a large repositioning of several sidechain oxygen ligands occurs only at the typical EF-hand, while the pseudo -EF-hand has minor structural changes upon Ca^2+^ binding [41]. Also, for S100A2 [42] and S100A5 [43] the presence of the ions did not change the shape of the CD spectra, but only discrete changes in ellipticity were observed at 195 and 208 nm. However, for S100A5, a huge change on its near-UV spectra was observed, thus suggesting that the effect of the ions was more pronounced on the tertiary structure of the protein. Unlike other members of S100 family, S100A10 does not use a calcium dependent mechanism in its interactions with target proteins [41].

The binding of recombinant porcine S100A12 to Ca^2+^, Zn^2+^, or Ca^2+^/Zn^2+^, caused only a minor spectral change in the SRCD spectrum of the protein. Very slight increases in the intensity of the peaks attributable to α-helical structures were observed upon binding to the ions (Figure 2). Similar behavior has been observed in the CD spectra of calbindin, another calcium binding protein, when bound to Zn^2+^ [44] and Ca^2+^ [45], where the secondary structure of the protein was not greatly affected. Very modest changes have also been observed in the three-dimensional structure of the apo-calcyclin (S100A6) upon Ca^2+^ binding [46], contrasting with the large structural changes caused by the opening of the globular domains in the Ca^2+^ sensors proteins.

**Figure 3:**
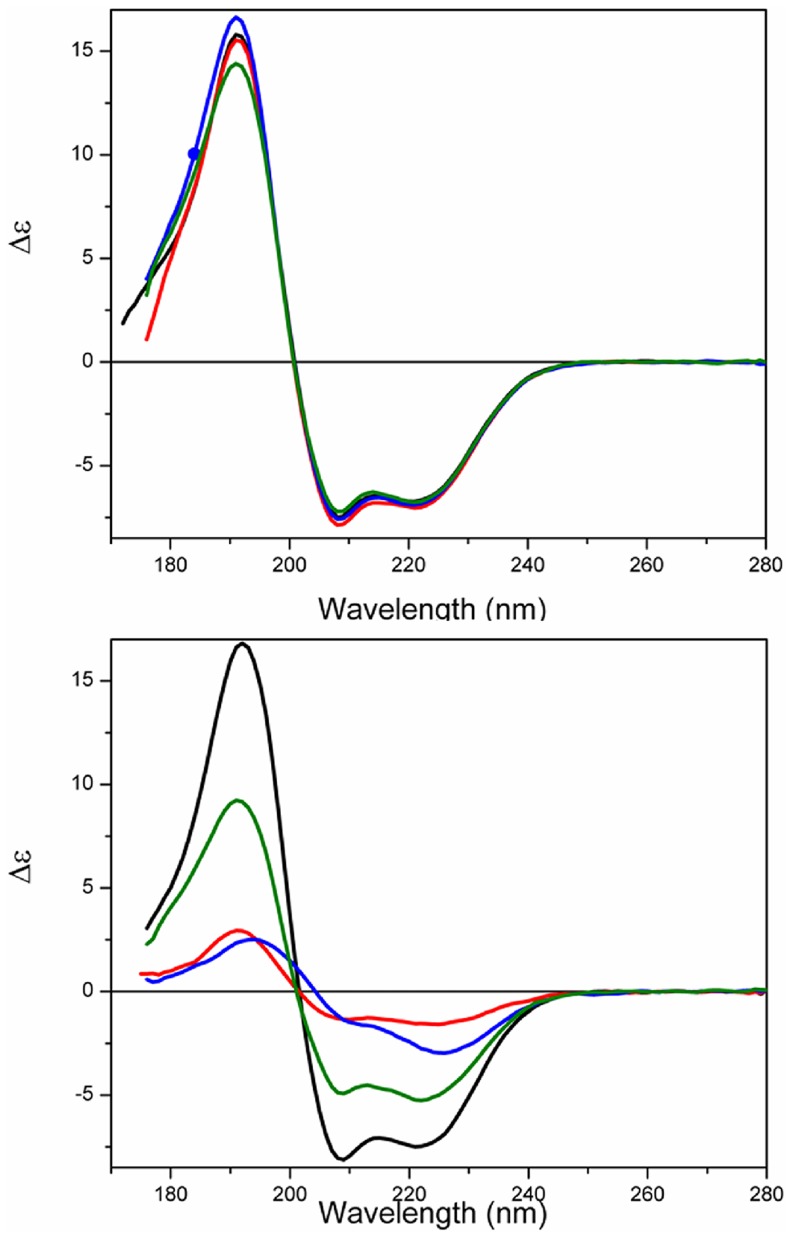
SRCD spectra of S100A12 in the presence of a) DPPC (black) and b) DPPG (black) without ions, and, in both cases, incubated with Ca^2+^ (red), Zn^2+^ (blue) or Ca^2+^/Zn^2+^ (green) together.

The SRCD spectra of S100A12 bound to the liposomes in the absence of the ions were not significantly different from those of the protein in aqueous solution (Figure 3), suggesting again that only minor conformational changes might occur in the overall secondary structure of the protein upon binding to the liposomes. The SRCD analysis suggests that the differences that had been observed in fluorescence intensity might be associated with local changes in the regions of the Tyr residues that do not affect the overall fold of the protein.

Although the SRCD spectra of the S100A12 bound to both types of liposomes are very similar, a slight red shift (∼2 nm) could be observed in the low wavelength region (below 205 nm) of the spectra in the presence of the DPPG liposomes. A small increase (∼5%) in the helix content was observed in the presence of the DPPG vesicles, which may reflect a slight ordering process resulting from positively charged residues being attracted to the oppositely charged vesicle surface.

The binding of S100A12 to liposomes in the presence of the ions showed dramatic differences depending on the surface charge of the vesicles. For the zwitterionic DPPC liposomes, only minor changes in the signal were observed in the presence of Ca^2+^, Zn^2+^ or both Ca^2+^/Zn^2+^ (Figure 3a). The calculated secondary structure content of the protein bound to the ions in the presence of DPPC showed no significant changes. Conversely, when S100A12 was bound to the negatively charged liposomes (DPPG), huge spectral changes were observed (Figure 3b) in the presence of the ions. The helical content of the protein was decreased to 30% or less when Ca^2+^ or Zn^2+^ were present. The binding of S100A12 to ions appears to facilitate its interaction with liposomes. This may be similar to the behavior observed for S100A13 [47], in which the binding to the ions promotes the availability of a solvent-exposed hydrophobic surface(s) in the protein, facilitating its interaction with lipid vesicles.

### Thermal Stability

**Figure 4:**
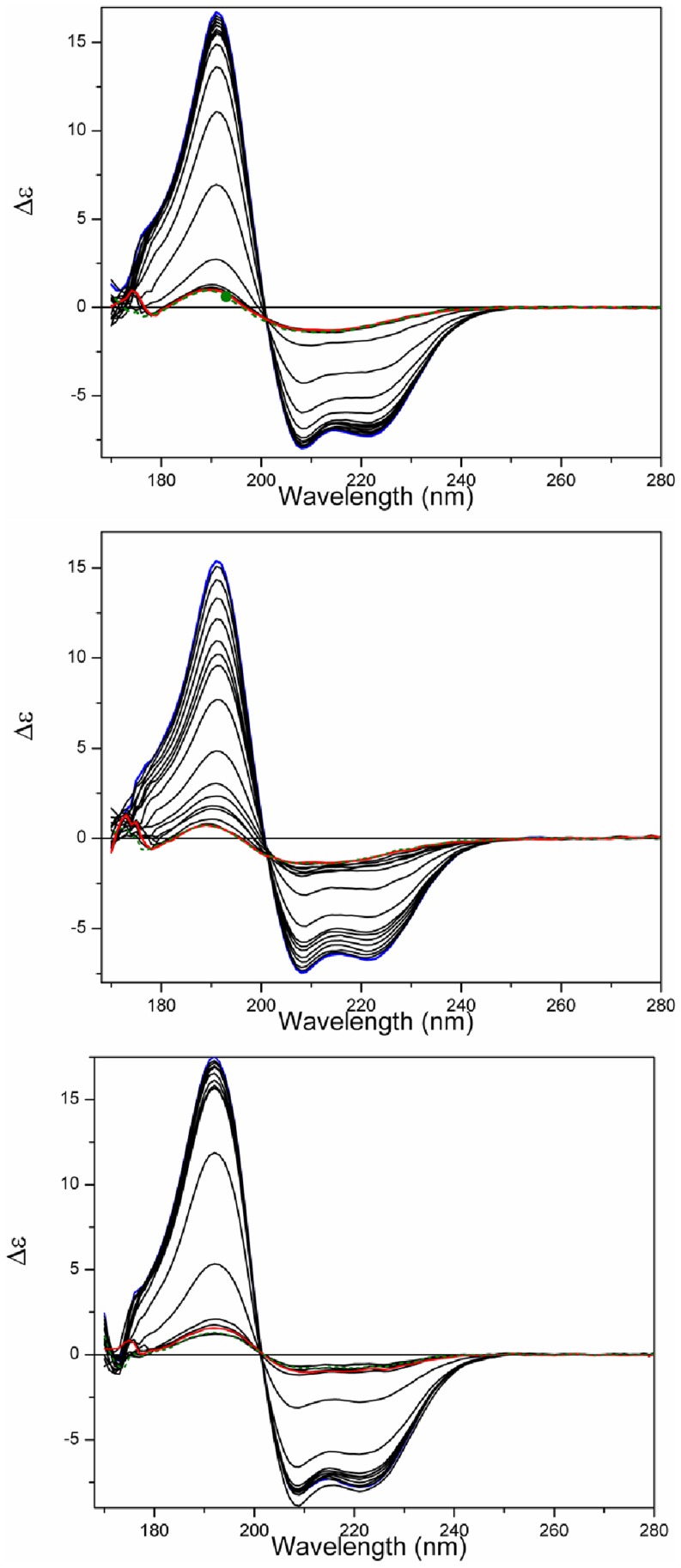
Thermal stability studies. a) SRCD spectra of S100A12 in the absence of vesicles or b) in the presence of DPPC vesicles or c) in the presence of DPPG vesicles as a function of temperature. The temperature range was from 5°C (blue) to 85°C (red), in 5°C steps (intermediate curves in black). After the heating process, the sample was cooled to 25°C (dashed green).

**Figure 5:**
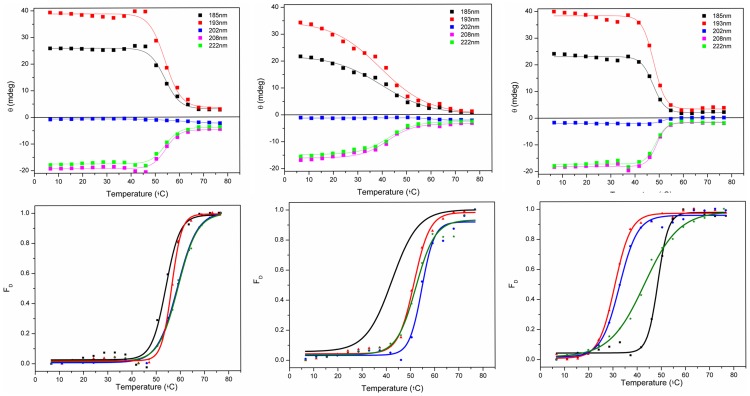
Curves monitoring the 185, 193, 202, 208 and 222) the absence of vesicles or in the presence of b) DPPC or c) DPPG vesicles. T_m_ determination for S100A12 denaturation in d) the presence of the ions or e) in the presence of the ions and DPPC vesicles, and f) in the presence of the ions and DPPG vesicles. Transitions were monitored at 222 nm and expressed as fraction of protein denatured (F_D_), using a sigmoidal curve fit on Origin software.

The temperature of melting (T_m_) determined for apoS100A12 in aqueous solution using SRCD was ∼54°C (Figure 4a, Table 2). The protein effectively retained its native state (as indicated by the intensities of the 222, 208, 193, and 185 nm peaks) up to 45°C, but beyond this point the thermal denaturation appeared to be a highly cooperative process (Figure 5a), with the protein assuming an essentially fully denatured state above 65°C. As the T_m_ values calculated based on all the different wavelengths (which monitor different secondary structural features) are fit by a single sigmoid-like function, this suggests that the unfolding may be a highly cooperative 2-state process, involving simultaneous changes throughout the entire molecule.

**Table 2:** Tm values for porcine S100A12 in the presence of liposomes, with and without Ca^2+^ and Zn^2+^.

Sample/Tm	No lipids (°C)	DPPC (°C)	DPPG (°C)
**No ion**	54.2±0.5	41.1±0.9	48.2±0.3
**Ca^2+^**	56.3±0.4	51.3±0.3	30.6±0.2
**Zn^2+^**	58.7±0.4	54.6±0.6	31.6±0.7
**Ca^2+^/Zn^2+^**	62.0±0.5	52.7±0.9	41.8±0.4

The binding of the S100 proteins to ions is known to enhance their thermal stability [48], which was also previously demonstrated for porcine S100A12 [21]. Although only minor changes were observed in the secondary structure of S100A12 when bound to the ions at 25°C, its thermal stability was significantly enhanced after binding to the ions, as seen from the increase of the T_m_ values of the holo- protein (Table 2).

The thermal stability of several members of the S100 family has been characterized by far-UV CD and differential scanning calorimetry [42], [49]–[51]. Table 3 shows the T_m_ values for some S100 proteins in the apo-state and for the heterodimer formed by the S100A8 and S100A9. Most of these proteins would be considered to be thermostable, with temperature of melting (T_m_) for the apo-form above 50°C. Similarly to S100A12, the thermal stabilities of these S100 proteins were increased in the presence of calcium. The effect of the distinct metal ions on the thermal stability of S100 proteins has demonstrated different behaviors. For S100A2, the ions Zn^2+^ and Ca^2+^ were shown to regulate protein thermal stability antagonistically, Zn^2+^ being a destabilizer and Ca^2+^ a stabilizer [42], however in the presence of both ions the thermal stability of S100A2 was also increased, in the same way to what is observed for porcine S100A12.

**Table 3:** Apparent Tm values for other members of S100 family from references [14]–[18].

Protein	Apo (°C)	Ca^2+^ (°C)	Zn^2+^ (°C)	Ca^2+^/Zn^2+^ (°C)
*S100A12*	54.2±0.5	56.3±0.4	58.7±0.4	62.0±0.5
*S100A2*	58.4	68.1	56.6	65.0
*S100A8*	59.9	65.8	-	-
*S100A9*	52.8	70.0	-	-
*S100A8/A9*	68.7	79.7	-	-
*S100A13*	76±1	92±1	-	-
*calbindin D9k (S100G)*	85	>100	-	-

Conversely, the thermal stability of porcine apoS100A12 in the presence of both vesicles of DPPC and DPPG (Figure 4b and 4c, respectively) was reduced. For the DPPC vesicles the cooperativity of the denaturation was also reduced (Figure 4e), while the protein bound to the negative liposome retained its high cooperative transition to a denatured state.

In the presence of the lipids and ions, two distinct behaviours were observed for the thermal stability of S100A12. The complete set of CD spectra and thermal transition curves for S100A12 in the presence of each of the ions and/or the vesicles are shown as Supplemmentary Figures. First, in the presence of the zwitterionic vesicles, the T_m_ values were enhanced when the protein was bound to Ca^2+^, Zn^2+^ or both ions together (Table 2), in a similar way to that observed in the absence of the lipids. In contrast, in the presence of the DPPG liposomes, the T_m_ values of S100A12 were dramatically decreased when the ions were added. A similar behavior was observed for S100A13 [47], in which the T_m_ for unfolding of the protein was decreased by ∼30°C in the presence of negatively-charged vesicles, showing the protein was severely destabilized upon interaction with the vesicles. It seems probable, therefore, that the formation of the S100A12-ion-liposome complex requires interactions of S100A12 with the head groups of the phospholipid via charged residues that might contribute significantly for ion binding. This additional interaction is probably affecting the residues involved in the binding of both metal ions and changing dramatically protein stability and folding.

### Surface Plasmon Resonance (SPR)

**Figure 6:**
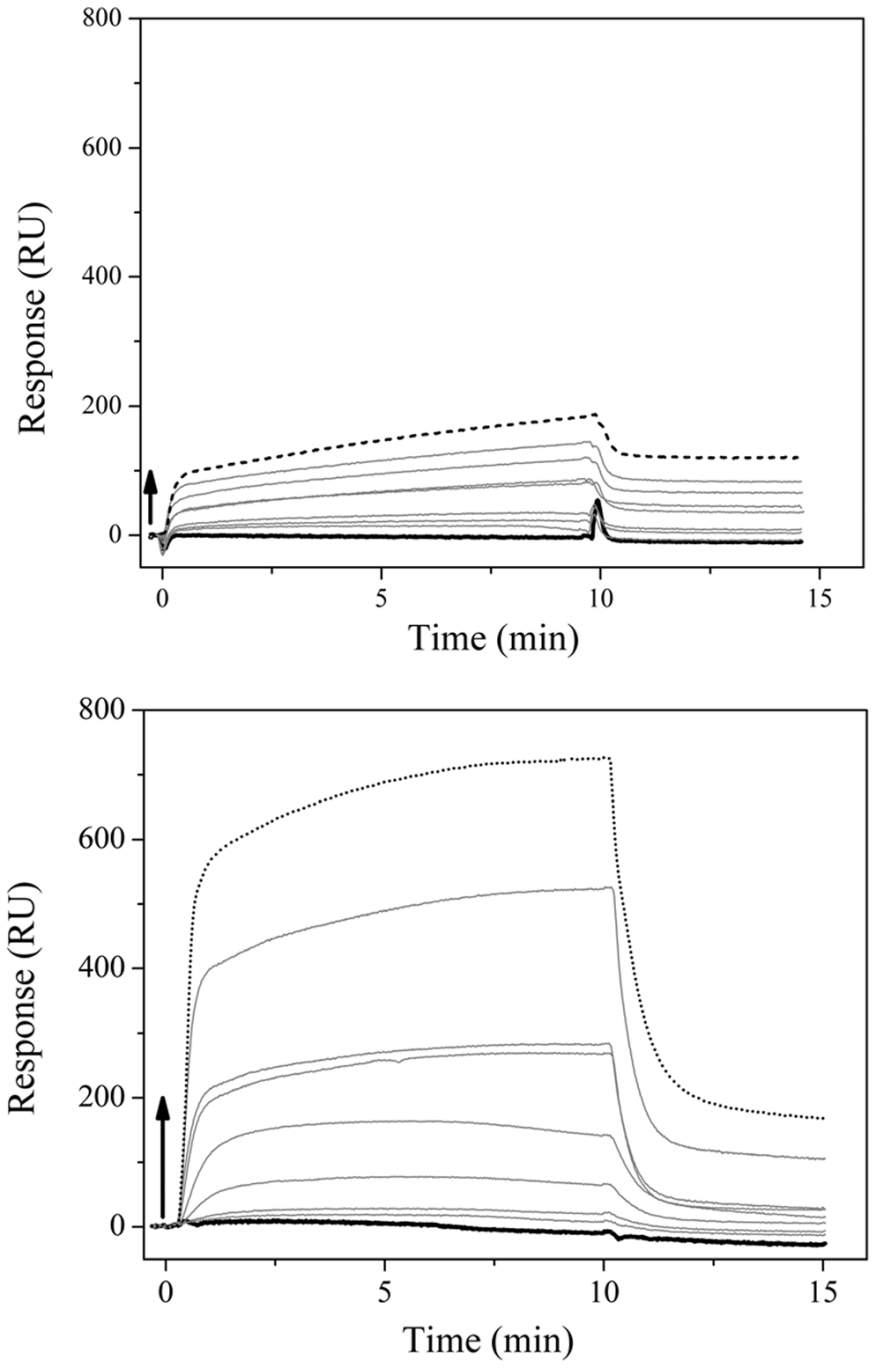
SPR sensorgrams of the porcine apoS100A12 adsorption onto a) DPPC and b) DPPG bilayers immobilized on a L1 sensorchip. Injections of the protein were made at 0 µL/min flow rate, at 25°C, for 10 minutes, and running buffer was added to start the dissociation. Arrows indicate the points where the S100A12 concentrations were increased: 31 nM (bold), 62 nM, 125 nM, 250 nM, 0.5 µM, 1 µM, 2 µM, 4 µM, and 8 µM (dot).

The SPR studies were used as complimentary evidence that S100A12 is able to bind to the liposomes in the apo-form and to characterize its apparent affinity toward the lipid bilayers. The interactions of S100A12 with DPPG and DPPC were observed on the surface of the L1 sensorchip. The sensorgrams for the interaction with the immobilized bilayers (Figure 6) showed that in both cases an increased of the amount of protein was bound to the lipids when increasing concentrations of protein were applied to the sensorchip. The behavior of the signal depended on the increase in concentration of the protein, and so enabled the determination of the affinity of the porcine S100A12 for the different types of phospholipid surfaces. The fits of the sensorgrams produced dissociation constants (K_d_) for DPPG and DPPC bilayers of 0.63 and 5.9 µM, respectively. The 10-fold increase for the DPPC lipids could be attributed to the role of electrostatic interactions in the protein-lipid binding, since S100A12 is positively charged (pI of 5.5) at neutral pH.

The kinetic parameters of the association and dissociation processes of S100A12 from the lipid bilayers are summarized in Table 4. The values determined are similar to those found for calponin [52] binding to liposomes of phosphatidyl serine (1.3 µM) and phosphatidyl inositol (1.5 µM).

**Table 4:** Association (k_a_) and dissociation (k_d_) rate, and dissociation constant (K_d_) of S100A12 on DPPC and DPPG bilayers immobilized on a L1 sensorchip.

Lipid	k_a_ (1/Ms).10^3^	k_d_ (1/s).10^−2^	K_d_ (µM)
**DPPC**	6.13	3.60	5.90
**DPPG**	15.5	0. 97	0.63

### Leakage Assays

The main purpose of the leakage assays was to investigate the perturbation caused on the vesicles due to the presence of the protein. We had investigated how the structure of the apo-S100A12 was affected after binding using SRCD and fluorescence studies, and here we intended to focus on the effects caused on the lipid system S100A12 was able to disturb the liposomes, releasing calcein (Suppl. Information). The extent of calcein release was proportional to the amount of S100A12 added. However, only a moderate leakage action was observed. 36% leakage was reached in the presence of 1 µM of S100A12 (corresponding to a ∼1∶10 protein-to-lipid molar ratio). This moderated leakage could be a result of the protein adsorption at the liposome surface, without an efficient penetration into the lipid acyl chains, in agreement with the fluorescence measurements with DPPC liposomes.

### Overall interaction mechanism between S100A12 and model membranes

Taken together, the SRCD results suggest that the binding of the ions to S100A12 in the presence of zwitterionic membranes does not significantly affect its secondary structure content and fold, although different lipid head groups do have significant influence on the protein stability. On the other hand, the changes observed in fluorescence suggest that tertiary changes, including the microenvironment of the Tyr residues, were influenced by the presence of the ions, and SPR studies indicate the relative strength of the lipid-protein interactions.

The higher affinity of S100A12 for negatively charged vesicles indicates that the charge interactions between the S100A12 and the liposomes surface can play a role in modulating the action of this protein onto biological membranes. This result suggests that wherever charges are present in the real cell membrane that could be the docking point for S100A12. This has an interesting biological implication since it has been proposed that segregated domains in the lipid membrane can indeed act as functional units to trigger signals through the membrane. Moreover, the different electrostatic potential generated by charged domains in the membrane could be the way used by the protein to find its partner receptor (RAGE receptor has been suggested as that partner to human S100A12) in the case a more specific binding mechanism takes place.

The decrease of the melting temperatures of S100A12 in the presence of negatively charged membranes suggests that the protein overall structure could be in a less ordered structure in the presence of the charged region of the membrane and this would facilitate lipid-protein interactions as shown by our SPR results. Thus, the events involving the transition to a more labile (or less ordered) structure, the exposition of a hydrophobic cleft and the protein preference for negatively charged membranes (or domains in these membranes) all suggest that the interactions with ions and liposomes might work as a molecular triggering mechanism for the protein to adopt the adequate conformation and to find its targets on the membrane surface.

Our combined use of these techniques demonstrated that S100A12 protein is capable of interacting both with lipids and with ions in solution, and has enabled examination of changes that occur at different levels of structure organization.

## Conclusions

A number of well-characterized proteins have been shown to exist in dynamic exchange between cytosolic and membrane-associated states, regulated by specific cellular signals [10]. In response to variations in the level of calcium, S100 proteins can interact with distinct target proteins and cellular compartments that are implicated in multiple intracellular and extracellular activities.

Whilst crystal structures of S100 proteins from various species have detailed the structures of the holo- and apo- enzymes, they have not shown the dynamics of the molecules in solution, nor their interactions with ions/phospholipid molecules. In this study we used SPR, fluorescence and SRCD spectroscopies to show that S100A12 interacts with both zwitterionic and negatively-charged membrane both in its apo- and holo- forms. Upon such interactions, not only are there changes in the polypeptide backbone structures, as determined from the SRCD spectra, but also intrinsic fluorescence changes suggest that modified tertiary interactions may also be occurring that change the relative environments of one or more Tyr residues. The interactions drastically affect the protein thermal stability in the different environments. The SPR measurements have shown that the protein binds more tightly to negatively-charged lipids. In the presence of the ions Ca^2+^ and Zn^2+^, the conformational changes occurred in S100A12 due to the interactions with membranes which strongly depend on the surface charge of the vesicle. This suggests stabilization of charged residues in protein structure via electrostatic interactions with lipid head groups could modulate the S100A12 interaction with membranes. Local accumulation of phospholipids with negatively charged head groups (such as inositols and/or phosphoglycerols) in the granulocytes membrane could provide the surface with electrostatics in which the interaction with S100A12 could take place. Taken together with the important role of Ca^2+^ as second messenger in various signaling pathways, and the remarkable interaction of S100A12 with membranes in the presence of these ions, it is probable, therefore, the translocation pathway of S100A12 might occur in response to an increase in intracellular levels of these ions, in a similar way as has been observed for other S100 proteins, such as S100A13 [53], S100A8 [54].

These studies of the molecular interactions, especially with lipid membranes, are thus complementary to the high-resolution crystal structure studies, and both types of information together can be optimally used to understand the molecular basis of the binding of lipids and divalent cations by S100A12. These in turn, provide insight into the roles of these proteins in molecular signaling and recognition.

## Supporting Information

Figure S1 a) SRCD spectra of the thermal stability study for S100A12 with Ca^2+^. The temperature range was from 5°C (blue) to 85°C (red), in 5°C steps (intermediate curves in black). After the heating process, the sample was cooled to 25°C (dashed green). b) Curve transition for S100A12 with Ca^2+^ monitored at 185, 193, 202, 208 and 222 nm peaks as a function of temperature.
(TIF)

Figure S2 a) SRCD spectra of the thermal stability study for S100A12 with Zn^2+^. The temperature range was from 5°C (blue) to 85°C (red), in 5°C steps (intermediate curves in black). After the heating process, the sample was cooled to 25°C (dashed green). b) Curve transition for S100A12 with Zn^2+^ monitored at 185, 193, 202, 208 and 222 nm peaks as a function of temperature.
(TIF)

Figure S3 a) SRCD spectra of the thermal stability study for S100A12 with Ca^2+^ and Zn^2+^. The temperature range was from 5°C (blue) to 85°C (red), in 5°C steps (intermediate curves in black). After the heating process, the sample was cooled to 25°C (dashed green). b) Curve transition for S100A12 with Ca^2+^ and Zn^2+^ monitored at 185, 193, 202, 208 and 222 nm peaks as a function of temperature.
(TIF)

Figure S4 a) SRCD spectra of the thermal stability study for S100A12 with Ca^2+^ in the presence of DPPC vesicles. The temperature range was from 5°C (blue) to 85°C (red), in 5°C steps (intermediate curves in black). After the heating process, the sample was cooled to 25°C (dashed green). b) Curve transition for S100A12 with Ca^2+^ in the presence of DPPC vesicles monitored at 185, 193, 202, 208 and 222 nm peaks as a function of temperature.
(TIF)

Figure S5 a) SRCD spectra of the thermal stability study for S100A12 with Zn^2+^ in the presence of DPPC vesicles. The temperature range was from 5°C (blue) to 85°C (red), in 5°C steps (intermediate curves in black). After the heating process, the sample was cooled to 25°C (dashed green). b) Curve transition for S100A12 with Zn^2+^ in the presence of DPPC vesicles monitored at 185, 193, 202, 208 and 222 nm peaks as a function of temperature.
(TIF)

Figure S6 a) SRCD spectra of the thermal stability study for S100A12 with Ca^2+^ and Zn^2+^ in the presence of DPPC vesicles. The temperature range was from 5°C (blue) to 85°C (red), in 5°C steps (intermediate curves in black). After the heating process, the sample was cooled to 25°C (dashed green). b) Curve transition for S100A12 with Ca^2+^ and Zn^2+^ in the presence of DPPC vesicles monitored at 185, 193, 202, 208 and 222 nm peaks as a function of temperature.
(TIF)

Figure S7 a) SRCD spectra of the thermal stability study for S100A12 with Ca^2+^ in the presence of DPPG vesicles. The temperature range was from 5°C (blue) to 85°C (red), in 5°C steps (intermediate curves in black). After the heating process, the sample was cooled to 25°C (dashed green). b) Curve transition for S100A12 with Ca^2+^ in the presence of DPPG vesicles monitored at 185, 193, 202, 208 and 222 nm peaks as a function of temperature.
(TIF)

Figure S8 a) SRCD spectra of the thermal stability study for S100A12 with Zn^2+^ in the presence of DPPG vesicles. The temperature range was from 5°C (blue) to 85°C (red), in 5°C steps (intermediate curves in black). After the heating process, the sample was cooled to 25°C (dashed green). b) Curve transition for S100A12 with Zn^2+^ in the presence of DPPG vesicles monitored at 185, 193, 202, 208 and 222 nm peaks as a function of temperature.
(TIF)

Figure S9 a) SRCD spectra of the thermal stability study for S100A12 with Ca^2+^ and Zn^2+^ in the presence of DPPG vesicles. The temperature range was from 5°C (blue) to 85°C (red), in 5°C steps (intermediate curves in black). After the heating process, the sample was cooled to 25°C (dashed green). b) Curve transition for S100A12 with Ca^2+^ and Zn^2+^ in the presence of DPPG vesicles monitored at 185, 193, 202, 208 and 222 nm peaks as a function of temperature.
(TIF)

Figure S10 a) SDS-PAGE of the purified S100A12. Column 1: molecular weight markers (mioglobin fragments), column 2: S100A12 obtained following gel filtration chromatography; b) Aligned sequences of human and porcine S100A12. Positively charged residues are in blue, negatively charged residues in red. Tyr residues are indicated by *, Ca^2+^ binding sites are underlined in grey (below the sequence) and residues which form the Zn^2+^ binding site in green (above the sequence).
(TIF)

Figure S11 Calcein leakage in DPPG liposomes promoted by the addition of S100A12. TritonX-100 (black) was used to reach 100% leakage. Protein concentrations were 1 µM (red), 5 µM (blue), and 10 µM (green).
(TIF)

Figure S12 Space filling of the homology models created for porcine apoS100A12 (first line), Ca^2+^- or Zn^2+^ bound form and for human S100A12 (second line), apo, Ca^2+^ and Zn^2+^-bound. Tyr18 is labeled in blue and the Tyr/Phe residue in the position 25 is labeled in cyan, respectively.
(TIF)
