# Supplementary figures and images for: Membrane Interactions of S100A12 (Calgranulin C)
Source: PLoS One. 2013 Dec 18;8(12):e82555. doi: 10.1371/journal.pone.0082555 (PMC3867360; doi:10.1371/journal.pone.0082555)

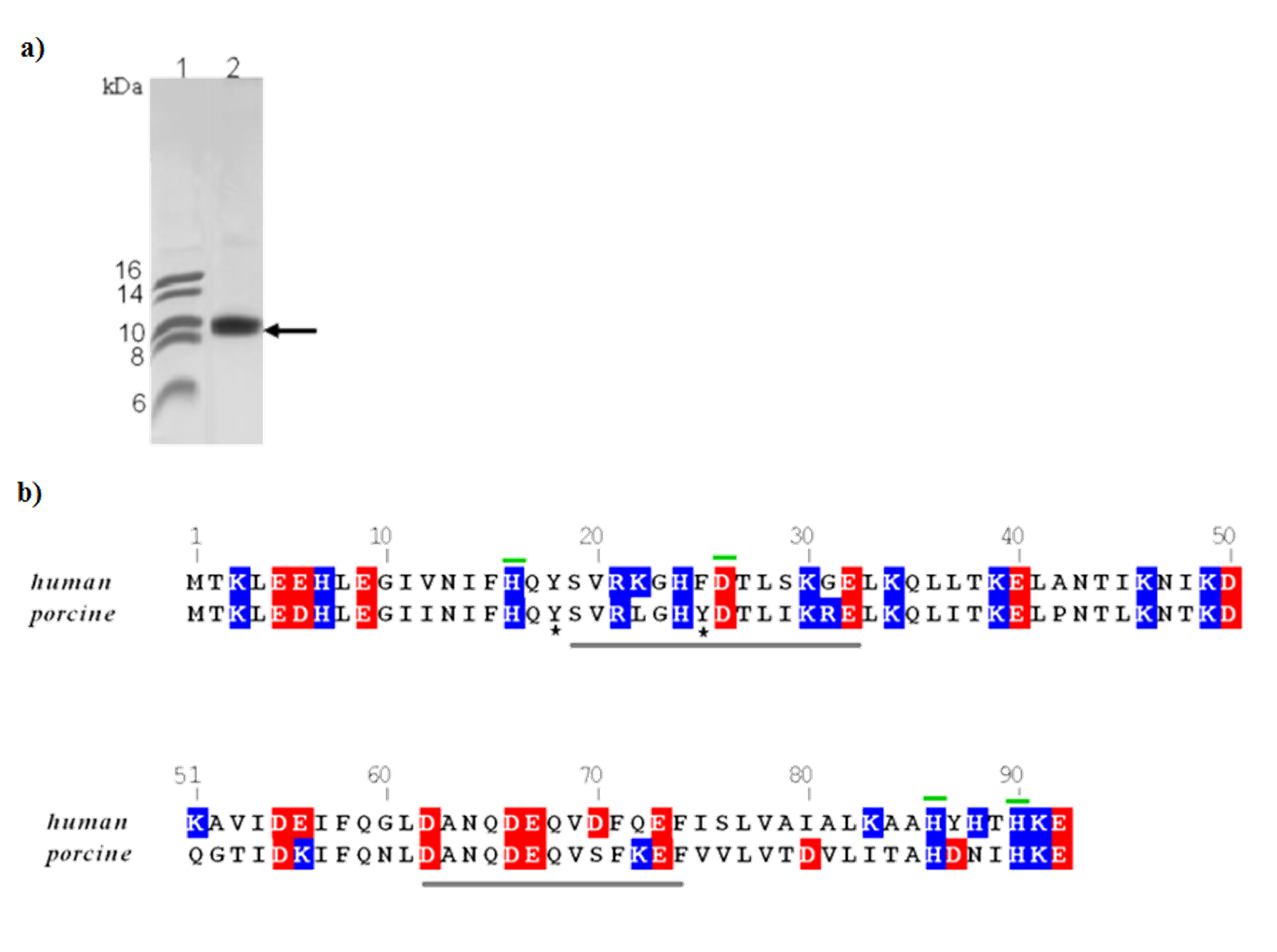

Supplement: Figure S10 — a) SDS-PAGE of the purified S100A12. Column 1: molecular weight markers (mioglobin fragments), column 2: S100A12 obtained following gel filtration chromatography; b) Aligned sequences of human and porcine S100A12. Positively charged residues are in blue, negatively charged residues in red. Tyr residues are indicated by *, Ca2+ binding sites are underlined in grey (below the sequence) and residues which form the Zn2+ binding site in green (above the sequence). (TIF) [file pone.0082555.s010.tif]

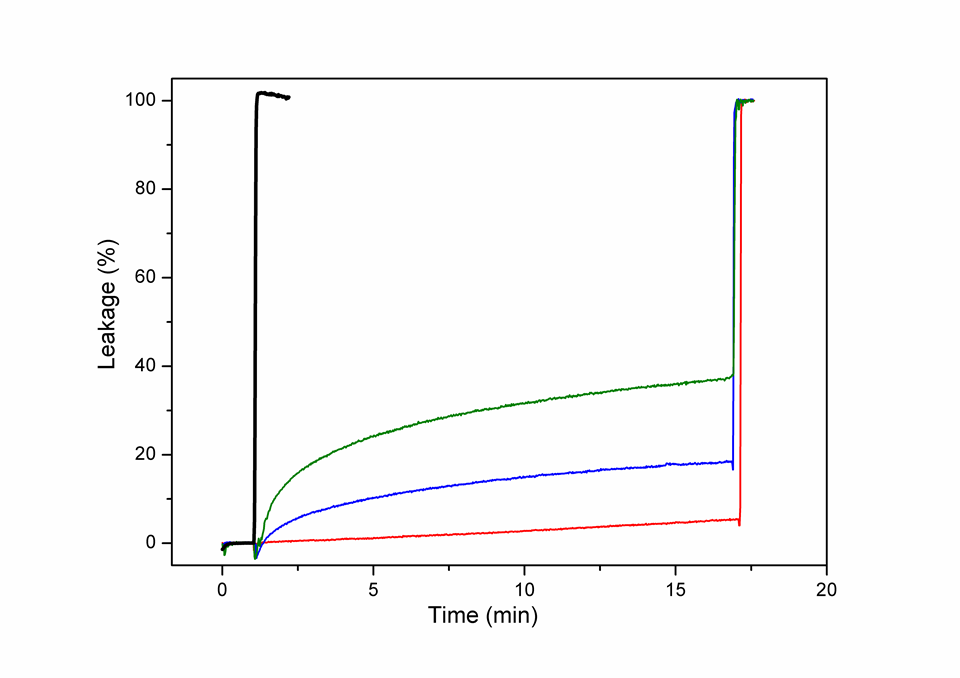

Supplement: Figure S11 — Calcein leakage in DPPG liposomes promoted by the addition of S100A12. TritonX-100 (black) was used to reach 100% leakage. Protein concentrations were 1 µM (red), 5 µM (blue), and 10 µM (green). (TIF) [file pone.0082555.s011.tif]

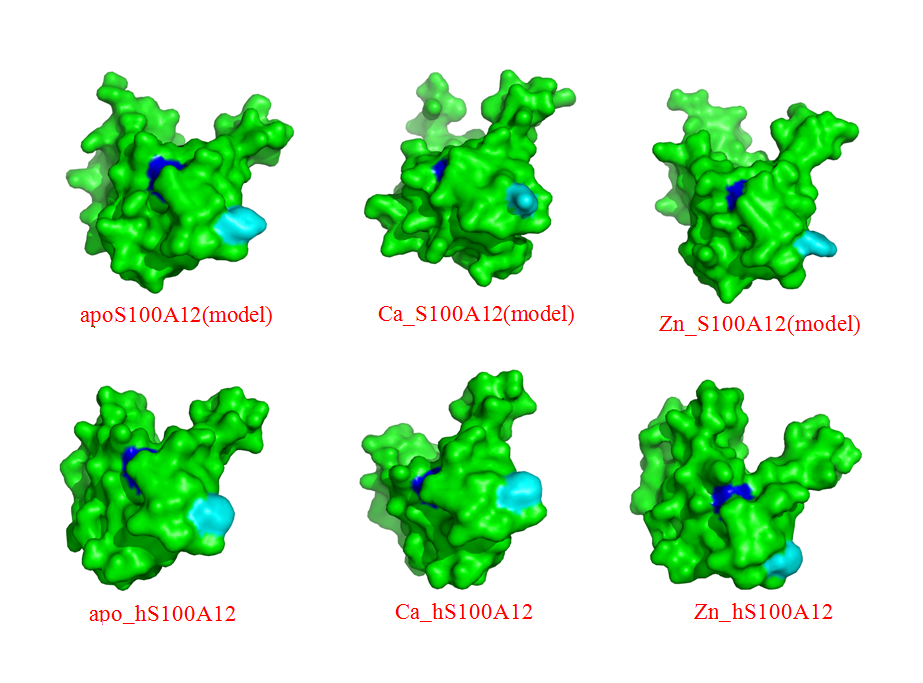

Supplement: Figure S12 — Space filling of the homology models created for porcine apoS100A12 (first line), Ca2+- or Zn2+ bound form and for human S100A12 (second line), apo, Ca2+ and Zn2+-bound. Tyr18 is labeled in blue and the Tyr/Phe residue in the position 25 is labeled in cyan, respectively. (TIF) [file pone.0082555.s012.tif]
